# Analyzing Event-Related Transients: Confidence Intervals, Permutation Tests, and Consecutive Thresholds

**DOI:** 10.3389/fnmol.2020.00014

**Published:** 2020-02-06

**Authors:** Philip Jean-Richard-dit-Bressel, Colin W. G. Clifford, Gavan P. McNally

**Affiliations:** School of Psychology, University of New South Wales, Sydney, NSW, Australia

**Keywords:** analysis, event-related transients, fiber photometry, family-wise error rate, bootstrap, permutation test

## Abstract

Fiber photometry has enabled neuroscientists to easily measure targeted brain activity patterns in awake, freely behaving animal. A focus of this technique is to identify functionally-relevant changes in activity around particular environmental and/or behavioral events, i.e., event-related activity transients (ERT). A simple and popular approach to identifying ERT is to summarize peri-event signal [e.g., area under the curve (AUC), peak activity, etc.,] and perform standard analyses on this summary statistic. We highlight the various issues with this approach and overview straightforward alternatives: waveform confidence intervals (CIs) and permutation tests. We introduce the rationale behind these approaches, describe the results of Monte Carlo simulations evaluating their effectiveness at controlling Type I and Type II error rates, and offer some recommendations for selecting appropriate analysis strategies for fiber photometry experiments.

## Introduction

**Figure 1:**
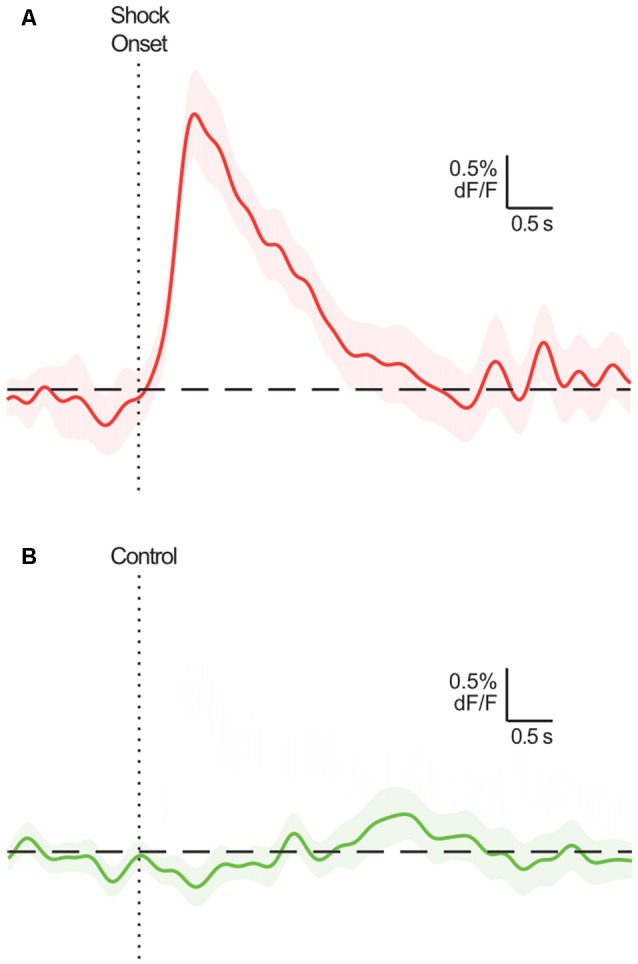
Example event-related calcium transients in BLA principal neurons (mean ± SEM; adapted from Sengupta et al., 2018; Figure 1C). These neurons exhibit a characteristic excitatory transient following shock delivery **(A)**, but not during a comparable period where the shock is not delivered **(B)**. Assuming the true response of these neurons is, in fact, excitatory for **(A)** and null for **(B)**, it would be optimal for statistical analyses to identify a significant excitatory transient at relevant time points in **(A)**, and no significant transients across the time points in **(B)**.

A broad objective for neuroscience involves identifying brain activity patterns and determining their function. The development of highly sensitive, novel fluorescent biosensors (e.g., calcium indicator GCaMP) and measurement techniques (e.g., fiber photometry) for use in the awake, freely moving animal have given behavioral neuroscientists powerful tools to chronically record neural dynamics of genetically- and circuit-defined populations *in vivo* (Gunaydin et al., 2014). Typically, the focus of this research is to determine whether there are phasic increases or decreases in activity around particular environmental and/or behavioral events, i.e., event-related activity transients (ERT). The presence of ERT implicates the targeted dynamic in a function related to that event, perhaps encoding signals that enable task-relevant perception, learning and/or behavior. Equally revealing is the types of events and situations that do not evoke ERT (Figure 1).

A widespread issue faced by researchers when using fiber photometry is how to best analyze the rich datasets they produce. A biosensor readout is a proxy for some underlying biological process (receptor binding, action potential, etc.,), so units of measurement are generally arbitrary. The recording time series is typically normalized into a delta F (dF) to represent relative activity change. Like all analysis strategies, the experimenter is confronted with a variety of choices such as whether to select these strategies before (*a priori*) or after (*post hoc*) data collection, how to avoid Type 1 (false positive) errors whilst achieving appropriate power to avoid Type II errors (false negative). To determine the presence of ERT, the dF around defined events can be collated and analyzed. The most common method involves obtaining a single number statistic quantifying a specific feature of the peri-event dF, such as the Area Under the Curve (AUC) or peak dF. This statistic is then used as input for null hypothesis tests, the results of which form the basis of interpretation (Gunaydin et al., 2014; Lerner et al., 2015; Sengupta et al., 2018).

Although simple and popular, the use of summary statistics such as AUC or peak dF raises concerns. This approach adds a cumbersome and problematic step to analysis: researchers choose the specific time window relative to events to summarize and analyze. If the window is too small, activity of interest is potentially missed; if the window is too large, the statistic loses meaningfulness (the temporal relationship between activity and event is undetermined). Even when a suitable window is chosen, results only reveal whether overall activity within the window is significantly different to the null, not where in this window activity is significant or whether activity beyond this window is significantly different from null. Therefore, using a summary of a time window discards potentially pertinent temporal information. Additionally, to minimize the probability of “missing” ERT, the analysis window is often chosen *post hoc*, after the experimenter has examined mean dF around an event. This is generally inconvenient, relatively arbitrary, and can introduce unwanted *post hoc* biases into the analyses, running the risk of significantly inflating the Type I error rate.

An alternative is to dispense with this kind of summary analysis to determine the presence of ERTs, and instead automatically analyze the entire peri-event period to determine whether, and when, a significant ERT occurs. This can overcome the limitations of AUC or peak dF approaches, but also raises its own concerns. For example, what kind of analysis is appropriate and how to effectively control the Type I error (false positive) rate whilst still achieving sufficient statistical power? Here we consider two straightforward alternatives to the use of summary statistics when analyzing fiber photometry data: (1) confidence intervals (CIs) around the peri-event dF waveform (e.g., Choi et al., 2019); and (2) permutation tests across the peri-event window (e.g., Pascoli et al., 2018).

### Confidence Intervals and Permutation Tests

A CI is a ranged estimate of a population parameter. In the case of mean peri-event activity, this would be a CI estimating the true population peri-event activity. Periods where the CI does not contain the null (e.g., *dF* = 0) can be flagged as significant, i.e., indicative of an ERT. In general applications, the parametric *t* interval (tCI) is most commonly used and is computationally simple to obtain: tCI = mean ± (SEM * *t*_crit_). However, a key assumption is that the underlying population distribution is normal, an assumption that may not be met by the recording data.

A non-parametric method to obtain CI is bootstrapping. Bootstrapping involves randomly resampling (with replacement) from the dataset and obtaining a bootstrap estimate from this sample. This is done repeatedly for all possible combinations of the dataset or a sufficiently high number of times (1,000 times or more). CIs can then be derived from the relevant percentiles of the resultant bootstrap distribution [percentile bootstrapped confidence interval (bCI); Efron and Tibshirani, 1993]. Importantly, this method makes no relevant assumptions about the underlying distribution and is more precise and accurate than tCI when using larger sample sizes. However, percentile bCIs have a narrowness bias for small sample sizes by an average factor of $\sqrt{(n-1)/n}$ (Hesterberg, 2015). This occurs because bootstrapping as an algorithm does not make any stipulations or adjustments related to *n*, leaving it open to issues related to small sample distributions. There are numerous ways to improve on these small sample properties (see Scholz, 2007; Hersterberg, 2014). A simple way is to expand the percentile bCI by a factor that accounts for *n*, as is done for tCI *via* SEM and *t*_crit_.

Permutation tests, like bootstrapping, are a non-parametric resampling-based method. They evaluate whether the distributions of the two groups of data are exchangeable. This is achieved by randomly regrouping the data to evaluate how unlikely the observed difference between groups was. The proportion of permutations that have a larger difference than the actual difference translates to the permutation *p*-value. If datasets are exchangeable, a large proportion of the permutations would produce a larger difference than that observed and the *p*-value would be large (i.e., insignificant). If the distributions are not exchangeable, it is unlikely for a random permutation to produce a larger difference, and the *p*-value may be critically small, leading to rejection of the null that datasets are exchangeable.

Permutation tests effectively produce an exact *p-*value, and can thus control Type I error at α, which is not true of percentile bCI. However, permutation tests do have some caveats. Permutation tests specifically concern distributions, not parameters. Permutation tests must compare two distributions (e.g., a peri-event sample vs. a baseline sample). Interpretation of significance must take into account that an effect may be driven by either distribution and may be caused by differences in the distributions beyond the parameter of interest (e.g., may be driven by differences in underlying population variances, not the population mean). They cannot be used to derive CI for a parameter such as mean dF (Hersterberg, 2014) and cannot test hypotheses regarding single-sample means (e.g., *dF* = 0). Finally, the level of significance detectable by permutation tests is constrained by the number of possible permutations and thus requires a minimum sample size for a given α (e.g., *n* = 4 has a minimum *p-*value of 0.014). That said, permutation tests are straightforward, have appealing statistical properties, and have been used to analyze peri-event neural activity (Maris and Oostenveld, 2007; Pascoli et al., 2018).

### Consecutive Thresholds

A key advantage of both CI and permutation tests is that they can be used to analyze peri-event activity by constructing a CI or performing a permutation test for each time point within the peri-event window. This dispenses with the need to choose a restricted window from which to obtain a summary statistic, as the entire peri-event dF can be analyzed for the presence of ERT. An additional benefit is that multiple ERT features, excitatory and/or inhibitory, can be identified using a single analysis without substantial input from the researcher.

However, testing each point of the peri-event window raises the problem of multiple comparisons. Given that a null signal is not static—it is composed of random fluctuations (i.e., noise)—the risk of producing a Type I error somewhere within the peri-event window will increase as the window size increases, inflating the family-wise Type I error rate (FWER) above the nominal rate, α. This could be dealt with by adopting a more conservative per comparison α to reduce the FWER (e.g., Bonferroni correction). However, a well-known issue with this approach is that it can be prohibitively conservative (Sedgwick, 2014), increasing the Type II error rate (failure to detect real differences). This can be particularly disadvantageous for fiber photometry, as sample sizes can be small (particularly when using subject-based analysis, as might be desirable in fiber photometry; (Recommendations … 2018) and the number of tests done across a peri-event window may render correction prohibitively conservative.

**Figure 2:**
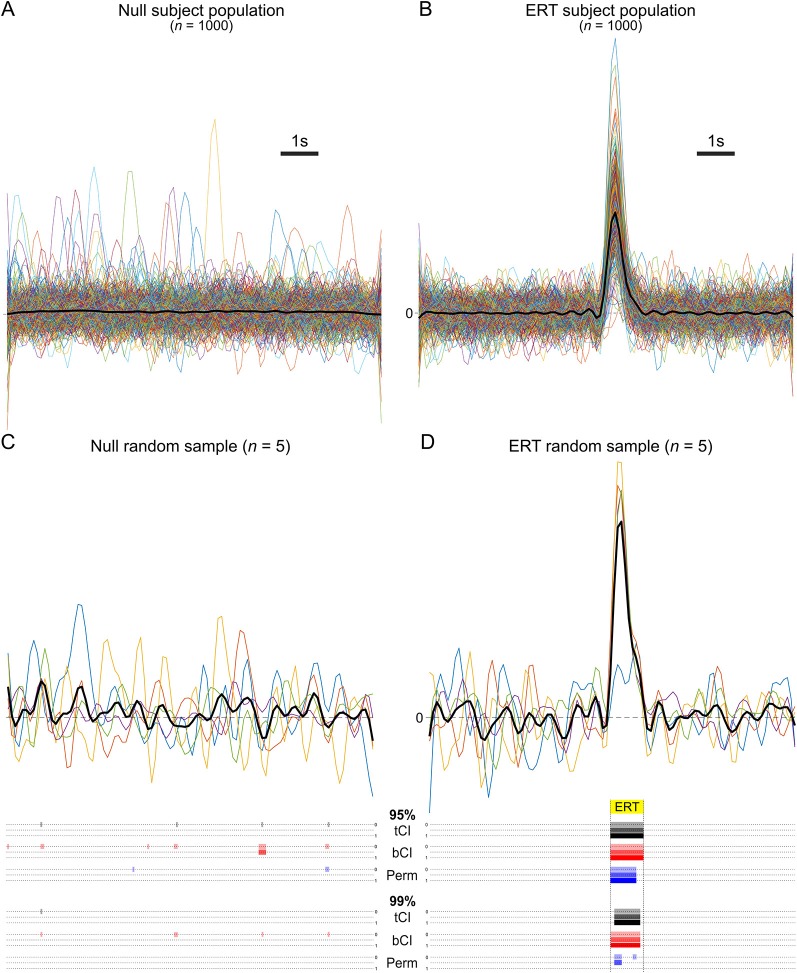
**(A,B)** Population of mean subject waveforms. **(C,D)** Example run [sample *n* = 5 (colored lines; mean = black line)] from Monte Carlo simulation and results of various analyses [parametric *t* interval (tCI), bootstrapped confidence interval (bCI), permutation test (Perm)] at 95% and 99% significance levels. **(C)** Example run when sampling from the null population. Colored bars beneath sample traces indicate where significant differences from null were incorrectly detected (Type I error), per analysis method. Without a consecutive threshold (top dotted line/colored bars per method), all analysis methods detected significant deviations from null at the 95% level; each produced Type I errors. A consecutive threshold (low-pass frequency window) prevented this error (bottom dotted line/colored bars per method). **(D)** Example run when sampling from event-related activity transients (ERT) population. Colored bars beneath sample traces indicate where in the ERT period (yellow highlighted period) significant differences from null were correctly detected, per analysis method. At the 95% level, tCI and bCI correctly rejected the null for the full extent of the ERT period, while the permutation test correctly rejected 80%. Consecutive thresholds did not impact this finding. At the 99% level, a smaller proportion of the ERT was identified. Importantly, the permutation test failed to meet the full low-pass consecutive threshold, and thus failed to identify a significant transient (Type II error).

Consecutive thresholds offer a simple, yet powerful, way to reduce Type I errors when detecting ERT, without commensurately increasing Type II errors. Consecutive thresholds demand a minimum period of continuous significance before accepting a transient as significant. The rationale here is that random fluctuations will generally produce Type I errors at the α rate, but are unlikely to do so for an extended period. This is intuitive when visualizing the interplay of random fluctuations and variance across the analysis window. The points of lowest variance will occur at time points when sample traces cross over (i.e., are equal). These cross-overs are inevitable and common for signals fluctuating around baseline, regardless of how signals are collated and averaged. Due to the low variance at these time points, they have low standard error and narrow CI that may not encompass the null. These cross-overs are generally a product of sample traces moving in opposite directions (e.g., one going up, the other going down) and thus represent a point, not an extended period, of low standard error (see Figure 2C). For an extended Type I error, the fluctuation must be aligned (i.e., be in phase), which is exponentially less likely, particularly as sample size increases. In contrast, ERTs by definition temporally coincide and are thus much more likely to yield a continuous period of significance. Adopting a moderate consecutive threshold, where the analysis demands a minimum period of continuous significance before accepting a transient as significant, can therefore greatly reduce Type I (and FWER) without necessarily affecting detection of true transients.

To evaluate the effectiveness of CIs, permutation tests and consecutive thresholds at controlling Type I and Type II error rates when detecting ERT, we assessed these methods in Monte Carlo simulations of artificially generated time-series datasets.

## Materials and Methods

Artificial datasets were generated and Monte Carlo simulations of analyses were conducted using custom MATLAB scripts (available at https://github.com/philjrdb/ERTsimulation).

Lines were generated for a null condition and ERT condition (*n* = 10,000 for each population type). Each line vector began as 100 zeros, representing a 10 speri-event baseline sampled at 10 Hz, to which Gaussian noise (10 dB) was added and low-pass filtered (2 Hz) to emulate randomly fluctuating noisy signal. A transient was operationalized as a one sparabolic curve (magnitude randomized using positive tail of *z* distribution). Each line in the ERT condition had a transient inserted at the halfway point of the window. Lines in the null condition had a 50% chance of a transient being inserted somewhere within the window, to emulate unrelated transients, or did not have any transient inserted. To emulate subject-based analysis, a subject population for each condition (*n* = 1,000; Figures 2A,B) was generated by randomly sampling and averaging 1–31 lines from their respective activity populations. These parameters correspond approximately to data generated by fiber photometry recordings (Sengupta et al., 2018; Choi et al., 2019).

The two key questions were: (1) How effective are tCI, bCI, and permutation tests in detecting the ERT when sampling from the ERT population, while retaining the null (not detecting a “transient”) when sampling from the null population? (2) What is the effect of applying a consecutive threshold to these results? We considered the effect of three different thresholds: no threshold (0), half the size of the low-pass frequency window (0.5), and the size of the low-pass frequency window (1). Half of the low-pass frequency window corresponds to the unidirectional component of noise at this threshold and therefore represents a cut-off for the most common source of Type I errors: unaligned high-frequency noise. However, this threshold is relatively lenient; phase-aligned noise and noise from slightly lower frequencies may still trigger Type I errors. An alternative threshold is the full length of the low-pass filter window. This window would more effectively remove Type I errors caused by high-frequency noise, like aligned random fluctuations, but could come at the expense of statistical power. A 2 Hz low-pass filter window is 0.5 s (1 s/Hz). Given that our simulation used a 10 Hz sampling rate, the $\frac{1}{2}$ consecutive threshold is three consecutive data points (rounded up) and the full consecutive threshold is five consecutive data points.

In total, we conducted 1,000 Monte Carlo simulations per sample size (*n* = 5–100). We randomly sampled *n* subjects from each subject population and analyzed them at 95% and 99% confidence levels, with and without consecutive thresholds (see Figures 2C,D). A tCI was calculated for each time point across the event window. For bCI, a bootstrap matrix of 1,000 bootstrapped means was acquired from *n* randomly resampled lines (with replacement). CI for each timepoint were percentiles at that timepoint of the bootstrap matrix (95%: 2.5, 97.5 percentiles; 99%: 0.5, 99.5% percentiles), which were then expanded by a factor of $\sqrt{n/(n-1)}$ to counter small sample narrowness bias. For either CI, a significant difference was flagged whenever the CI did not contain the null of 0 (Bird, 2004; Bland and Altman, 2015). Permutation tests require a comparison distribution, so ERT and null condition samples were tested against another random sample from the null population to represent a baseline comparison (new baseline comparison per simulation). All possible permutation or 1,000 random permutations, whichever was fewer, was used. The *p*-value for a time point was the proportion of permutations whose mean difference values were more extreme than that observed between the actual samples. A time point was flagged as significant if *p* < α.

The critical measures were FWER under the null condition and the correct reject rate for the ERT condition. FWER was determined as the proportion of null sample simulations that produced a significant effect (Type I error) within the peri-event window. Each simulation either had or did not have a Type I error. The correct reject rate was the proportion of the 1 sERT identified as significant per simulation; each simulation rejected 0–100% of the ERT.

Lastly, we use tCI, bCI and permutation tests to analyze the exemplar ERT and null data in Figure 1 (from Sengupta et al., 2018). The exemplar ERT data is of CS+ offset (coincident with shock delivery) trials on day 1 of fear conditioning (*n* = 23), whereas exemplar null data is of CS− offset (no shock comparison) trials on day 3 of fear conditioning (*n* = 24)^1^. tCI and bCI were used to determine the presence of ERT within each peri-event period (null: dF/F = 0). To demonstrate a relevant extension of these analyses, these peri-event waveforms were also compared against each other using the two-sample *t*-test and bootstrap (bootstrap difference distribution of randomly resampled means; Hersterberg, 2014). As permutation tests can only compare two samples, permutation tests were used to compare CS+ and CS−. A consecutive threshold equalling the low-pass frequency (3 Hz) window (1/3 s was applied to control FWER.

## Results

**Figure 3:**
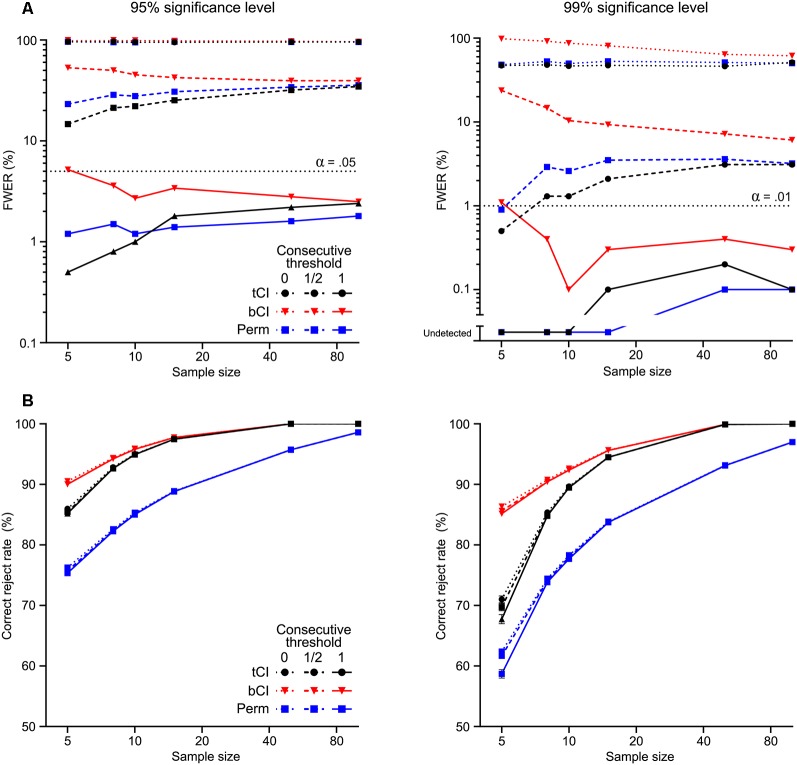
Performance of parametric *t* interval [tCI (black)], bootstrapped CI [bCI (red)] and permutation test [Perm (blue)] across 1,000 Monte Carlo simulations at various sample sizes (*n* = 5–100). Each simulation was tested at two significance levels [95% (left), 99% (right)]. **(A)** Family-wise Type I error rate (FWER) was determined as any false rejection of the null across the time window (i.e., detection of a “significant transient” from signals drawn from the null population). Each method had extremely high false-positive rates without a consecutive significance threshold (=0), regardless of the significance level. Use of a consecutive threshold substantially reduced FWER; a threshold set at the low-pass frequency (=1) reduced FWER to at or below nominal rates (α). **(B)** Correct rejection rate was determined as proportion of the artificial transient that was identified as non-zero. Bootstrapped CI was the most powerful, while permutation tests were the least powerful at detecting the extent (and presence) of the transient. Consecutive thresholds did not substantially reduce power to detect this transient.

The summary of results from the 1,000 simulations per selected sample sizes are shown in Figure 3. Without a consecutive threshold, all analysis methods were likely to detect a significant “transient” somewhere within the peri-event window, despite drawing from the null population (Figure 3A). In other words, the actual FWER was extremely high without a consecutive threshold, regardless of *n* or confidence level. The use of a consecutive threshold substantially reduced the FWER. A $\frac{1}{2}$ threshold substantially reduced FWER, although not to nominal rate, α. A full low-pass threshold reduced FWER to at or below nominal rate, α.

Regarding detection of the ERT (Figure 3B), each analysis method generally detected large proportions of the transient across simulations (on average >50%), with detection rate improving as sample size increased. Importantly, the use of consecutive thresholds had little impact on this. The bCI appeared to have the highest correct reject rate, while permutation tests had the lowest. This sensitivity was reflected in the likelihood for these methods to detect the ERT at all (i.e., whether a significant difference was detected within the ERT period or not). The bCI almost always correctly rejected the null—it only failed to detect the ERT <1% of simulations for *n* = 5, 99% confidence level, full consecutive threshold (no failure to reject for any other parameters). In contrast, permutation tests were most likely to miss rejecting the null, doing so under various conditions when *n* < 15 (for *n* = 5, 99% confidence level, full consecutive threshold, almost 10% of simulations failed to detect the ERT). It is worth noting here that previous applications of permutation tests to detecting ERTs (Pascoli et al., 2018) analyzed trials, not subject means. The choice of trials, rather than subjects, as the basis for the analysis of fiber photometry data, provides substantially larger *n* for analyses but raises independent, non-trivial concerns about correlations among the data (see Recommendations … 2018).

**Figure 4:**
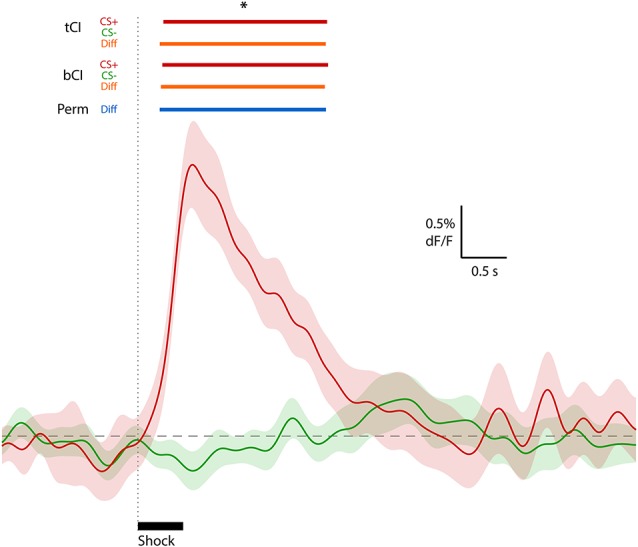
Waveform analysis of real peri-shock (CS+; red waveform) and control (CS−; green waveform) data depicted in Figure 1 (from Sengupta et al., 2018; Figure 1C). An excitatory ERT following shock onset for CS+ (red bars above graph), but no ERT for the CS− comparison, was identified using *t* interval (tCI) and bCI. A significant difference between CS+ and CS− (two-sample comparison) was also determined using these CI methods (orange bars above graph) and permutation tests (blue bar above graph). *Significant at 95% confidence level (full low-pass consecutive threshold).

To illustrate the effectiveness of these methods on real data, we applied them to the exemplar data depicted in Figure 1 (from Sengupta et al., 2018). Both tCI and bCI readily identified a significant excitatory ERT (relative to null of dF/F = 0) following shock delivery (Figure 4). The same analysis of CS− (no shock delivery) did not identify any event-related changes in the activity. Direct comparison of CS+ and CS− activity *via* two-sample tCI, bCI and permutation tests revealed that these traces significantly differed from each other for the duration of the identified shock ERT.

## Discussion

Identifying event-related transients (ERT) is a common focus of neural recording studies. A common approach to detecting ERT involves obtaining a summary statistic from a *post hoc*-specified period relative to an event (e.g., AUC). This introduces a problematic step within analysis. The current study considered alternative strategies that instead analyze the entire peri-event period for ERT: CIs and permutation tests. The effectiveness of these analyses, in combination with consecutive thresholds, at controlling Type I and Type II error rates was assessed in Monte Carlo simulations of artificial datasets that approximate fiber photometry data.

We found that CI or permutation tests can be effectively used in combination with consecutive thresholds to analyze peri-event periods for significant ERT. Both approaches afford good control over the Type I error rate; rates of inappropriate ERT detection (null condition) were acceptably low across *n* when using a consecutive threshold equalling the low-pass frequency window. Both also provided reasonable statistical power. In both cases, correct rejection of the null in the ERT condition was high, increasing with *n*, and was largely unaffected by the consecutive thresholds. Of the methods considered here, the bCI appears to be the most sensitive. The bCI had the highest likelihood of rejecting the null, especially at smaller sample sizes (*n* < 20), whereas permutation tests were the least sensitive. These differences between bCI and permutation tests narrowed as *n* increased, with little difference between bCI and permutation tests at *n* > 40. Compared to permutation tests, tCI had good properties, with similar FWER rates and fewer Type II errors.

When applied to real data, CI methods readily identified the extent of excitatory transients in amygdala to footshock, while showing a comparison shock-free period was not associated with changes in amygdala activity. We also compared these peri-shock and peri-control signals using two-sample CI methods and permutation tests to show the extent those signals diverged. These results demonstrate that these methods can effectively assess and convey the significance of peri-event activity change, and are amenable to making pertinent comparisons between signals (e.g., those around different events). Permutation tests are limited to two-sample comparisons, but CI methods could theoretically be applied within more complex analyses (e.g., polynomial contrasts) to assess diversity of questions regarding neural activity. However, there remain several issues that should be considered when analyzing photometry data, which we discuss below.

### Considerations for Analysis

#### Choosing the Analysis Procedure

There are a variety of options for analyzing fiber photometry data that each have merits and drawbacks (Table 1). Although the ideal analysis will depend on a researcher’s needs, we argue here that detection and comparisons of ERT is achieved more efficiently and effectively using waveform analysis methods. Waveform analyses automatically detect the extent of significant transients, whereas summary analyses typically require inconvenient and problematic *post hoc* input while discarding temporal information. However, a summary statistic may still be preferable when using peri-event activity in analyses that are overly complicated by the waveform vector. For instance, it is simpler to assess and convey the correlation between behavior and AUCs (Choi et al., 2019) than behavior and waveforms.

**Table 1:** Overview of transient analysis methods.

	Temporally-defined transient	Non-parametric	Single mean comparison?	Exact *p* value*	Range estimate	Pros	Considerations
**Summary analyses**			
AUC	×	Possible	✓	✓	Possible	• Simple	Undefined extent/location of transient Jittered transient still detected Issues with defining analysis window
Features (peak dF, peak frequency)	×	Possible	×	✓	Possible	Intuitive Analysis of various features	Requires a comparison period Issues with defining analysis window
**Waveform analyses**				
*t* interval	✓	×	✓	✓*	✓	• Simple	• Parametric assumption
Bootstrap interval	✓	✓	✓	×*	✓	Powerful Few assumptions	Computationally demanding compared to tCI Percentile interval does not inherently control per comparison Type I error rate at α
Permutation tests	✓	✓	×	✓*	×	• Conservative	Lowest power of methods considered Computationally demanding compared to tCI Requires a comparison distribution Distribution difference only —cannot directly indicate parameter range/direction

*Per comparison p value—diverges from alpha when using consecutive thresholds. Note. AUC, area under the curve; tCI, t interval.

A major conclusion of the current study is that consecutive thresholds effectively reduce the Type I error rate in waveform analyses without commensurately impacting ERT detection. This duration requirement is a simple but blunt way to filter out the most common source of Type I error—brief blips of significance due to chance alignments in high-frequency noise. ERT by definition align for extended periods of time and are thus less affected. However, consecutive thresholds can increase the Type II error rate, particularly if the threshold is overly conservative or the ERT very brief. It is therefore important to choose a threshold that will efficiently reduce FWER without undermining detection of ERT. The right threshold will depend on the temporal dynamics of neural activity and biosensor, as well as the properties of the signal being analyzed. We discuss these considerations in turn and explain why a consecutive threshold based on the low-pass filter window is a decent rule-of-thumb.

Of the most commonly used biosensors, GCaMP6f has the fastest dynamics and is thus more vulnerable to Type II errors from consecutive thresholds. Like most biosensors, GCaMP6f acts like a leaky integrator, such that its output over time is a decaying compound of inputs (Chen et al., 2013). This means the duration of a transient, not just the magnitude, is proportional to activity change. For instance, GCaMP6f dF/F is elevated (20%-peak) for ~0.4 s following a single action potential, but this duration is multiplied by the number of action potentials that occur within that window; a sub-second burst of population activity can produce a prolonged multi-second transient (Chen et al., 2013). This means ERT detection is resilient to consecutive thresholds in proportion to the effect size of activity change.

Regarding Type I errors, the effectiveness of a consecutive threshold in reducing FWER depends on the degree of high and low-frequency noise in the signal. As stated previously, high-frequency noise is responsible for frequent but brief instances of Type I errors, which consecutive thresholds effectively counteract. Type I errors due to chance alignments in low-frequency noise is less likely, but have a higher chance of lasting for extended periods, and are thus more resilient to consecutive thresholds. In fiber photometry, high-frequency components (>10 Hz) are typically attributed to electrical noise and are thus low-pass filtered out. The low-pass filter frequency is usually chosen based on the cut-off between signals of interest (e.g., temporal dynamics of the biosensor) and noise present in the signal, and therefore represent a natural cut-off for ERT vs. noise. Additionally, low-pass filters reduce the power of high-frequency components of a signal, allowing low-frequency components to dominate, which increases the autocorrelation of noise and the likelihood of extended Type I errors. Setting the consecutive threshold to the low-pass filter period is a way to peg the threshold to a factor that increases the need for a more conservative threshold.

It is also worth noting consecutive thresholds reduce FWER independently of per-comparison α. It, therefore, diverges from standard conceptions of α and *p*-values. If future studies ascertained the precise relationship between FWER, α and consecutive thresholds, the relevant equation could be used to calculate exact *p*-values, or be used to fine-tune the consecutive threshold for a given α and FWER. Such an equation would capture the likelihood of consecutive Type I errors—errors become exponentially less likely as the consecutive threshold increases (α^threshold^) unless there is a high degree of autocorrelation across that period. Autocorrelation of noise tends to decrease across time, so increasing the consecutive threshold also reduces this effect of autocorrelation. Given a fixed peri-event window, the threshold also decreases the number of consequential data points (i.e., degrees of freedom). Increasing the peri-event window (i.e., increasing the number of comparisons) increases FWER, although in a less substantial way than consecutive thresholds.

Finally, it is important to state that comparisons between event signals, as was done on the data in Figure 4, can be valid but should be done thoughtfully. Fiber photometry depends on a population-level biosensor readout. This readout depends on biosensor expression and fiber placement, which inevitably differ between subjects, making between-subject comparisons controversial. Furthermore, biosensor expression is dynamic, generally increasing over days and weeks, while recording causes bleaching of biosensor fluorophores within-session, making within-subject comparisons across time similarly contentious. Appropriate normalization of signal (see below) combats these influences but cautious interpretation remains warranted.

#### Interpreting Significance

Waveform analyses provide temporally-defined significance. Interpretation should factor in the relationship between the biological process of interest and biosensor readings. For instance, calcium-indicator (e.g., GCaMP) readout is a common proxy for neural firing rates. Signals from GCaMP are slightly lagged relative to spiking activity and have non-trivial decay times (Chen et al., 2013). The resultant leaky-integrator readout has two repercussions for interpretation: (1) an identified ERT is likely due to more confined, slightly earlier changes in underlying neural activity; and (2) the temporal extent of an ERT can be affected by both the magnitude or duration of activity change. This limits highly specific inferences about neural activity and the duration of ERT, although more general inferences about the anticipatory or deliberative activity when an ERT precedes event onset remain valid.

Both summary and waveform CI methods allow for single-mean comparisons. That is, the presence or absence of ERT can be inferred by testing against a specified null (e.g., dF/F = 0). This raises the important issue of having a valid null when using this method. Typical calculations for dF/F (e.g., subtraction of fitted isosbestic from the calcium-dependent signal; Lerner et al., 2015) normalizes the signal, giving it a mean of zero across the period that was used to calculate dF/F. However, trends may still exist in the data (e.g., a general decrease in a signal across the session) that impact peri-event signals and analysis. A common means to combat these trends are to detrend dF/F and/or zero peri-event signals to a pre-event baseline (Lerner et al., 2015; Pascoli et al., 2018; Sengupta et al., 2018; Choi et al., 2019). Although putatively effective, it remains important to consider the potential unintended effects of normalization and interpret accordingly (e.g., effects are relative to a particular baseline). Choosing an appropriate null will depend on the dataset and procedures applied, but it stands to reason that appropriate normalization and detrending render null of dF/F = 0 valid.

## Conclusions and Recommendations

There are a variety of options for analyzing fiber photometry data. Each of the methods described here has merits (Table 1). The waveform analyses we have considered here offer key advantages over summary analyses and the key recommendation from our findings is to use a sufficient consecutive threshold to reduce FWER when using these waveform analyses. The ideal threshold would consider the temporal dynamics of the specific biosensor being used (i.e., duration of transients), noise present in the recording signal, and the size of the peri-event window. A reasonable, effective rule-of-thumb is to apply a threshold equalling the low-pass frequency period, which itself should reflect the temporal cut-off between actual transients (i.e., at minimum, the temporal dynamics of the biosensor) and noise.

## Data Availability Statement

The datasets generated for this study are available on request to the corresponding author.

## Author Contributions

PJ and CC contributed to programming and statistical analysis. PJ and GM wrote the first draft of the manuscript. All authors contributed to manuscript revision.

## Conflict of Interest

The authors declare that the research was conducted in the absence of any commercial or financial relationships that could be construed as a potential conflict of interest.

## Footnotes

^1^Different days were used because waveforms from other days were less exemplary, making them less suitable for the current purpose of demonstration. For instance, activity for Day 1 CS− trended negatively (apparent in AUC; Sengupta et al., 2018).
